# 4-(1*H*-Tetra­zol-5-yl)benzene-1,3-diol

**DOI:** 10.1107/S160053681300411X

**Published:** 2013-02-16

**Authors:** Youngjo Kim

**Affiliations:** a Department of Chemistry, Chungbuk National University, Cheongju, Chungbuk 361-763, Republic of Korea

## Abstract

In the title compound, C_7_H_6_N_4_O_2_, rings are almost coplanar, the dihedral angle between them being 8.45 (13)°. An intra­molecular N—H⋯O hydrogen bond occurs. In the crystal, the mol­ecules are linked by O—H⋯N and N—H⋯O hydrogen bonds into a three-dimensional network.

## Related literature

For the structure of 4-(5-tetra­zol­yl)-1,3-benzene­diol sesquihydrate, see: Gallardo *et al.* (1995 ▶). For the synthesis, see: Meyer *et al.* (1998 ▶).
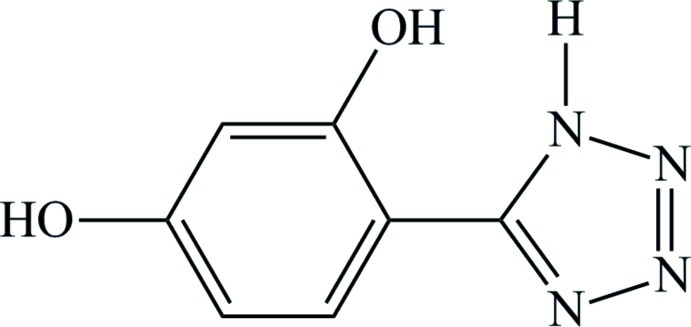


## Experimental

### 

#### Crystal data

C_7_H_6_N_4_O_2_
*M* *_r_* = 178.16
Orthorhombic, 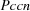

*a* = 16.109 (2) Å
*b* = 7.2931 (11) Å
*c* = 12.8708 (17) Å
*V* = 1512.2 (4) Å^3^
*Z* = 8
Mo *K*α radiation
μ = 0.12 mm^−1^
*T* = 296 K
0.10 × 0.10 × 0.08 mm

#### Data collection

Bruker APEXII CCD diffractometer
Absorption correction: multi-scan (*SADABS*; Bruker, 2006 ▶) *T*_min_ = 0.96, *T*_max_ = 0.98
20096 measured reflections
2452 independent reflections
1273 reflections with *I* > 2σ(*I*)
*R*_int_ = 0.110

#### Refinement

*R*[*F* ^2^ > 2σ(*F* ^2^)] = 0.057
*wR*(*F* ^2^) = 0.168
*S* = 1.05
2452 reflections
124 parameters
H atoms treated by a mixture of independent and constrained refinement
Δρ_max_ = 0.35 e Å^−3^
Δρ_min_ = −0.26 e Å^−3^

### 

Data collection: *APEX2* (Bruker, 2006 ▶); cell refinement: *SAINT* (Bruker, 2006 ▶); data reduction: *SAINT*; program(s) used to solve structure: *SHELXS97* (Sheldrick, 2008 ▶); program(s) used to refine structure: *SHELXL97* (Sheldrick, 2008 ▶); molecular graphics: *ORTEP-3 for Windows* (Farrugia, 2012 ▶); software used to prepare material for publication: *SHELXTL* (Sheldrick, 2008 ▶).

## Supplementary Material

Crystal structure: contains datablock(s) I, global. DOI: 10.1107/S160053681300411X/ff2096sup1.cif

Supplementary material file. DOI: 10.1107/S160053681300411X/ff2096Isup2.cdx

Structure factors: contains datablock(s) I. DOI: 10.1107/S160053681300411X/ff2096Isup3.hkl

Supplementary material file. DOI: 10.1107/S160053681300411X/ff2096Isup4.cml

Additional supplementary materials:  crystallographic information; 3D view; checkCIF report

## Figures and Tables

**Table 1:** Hydrogen-bond geometry (Å, °)

*D*—H⋯*A*	*D*—H	H⋯*A*	*D*⋯*A*	*D*—H⋯*A*
O1—H1⋯N4^i^	0.82	1.94	2.759 (3)	173
O2—H2⋯N3^ii^	0.82	2.00	2.817 (3)	173
N1—H101⋯O1	0.89 (3)	2.22 (3)	2.701 (3)	113 (2)
N1—H101⋯O2^iii^	0.89 (3)	2.40 (3)	3.034 (3)	129 (2)
C3—H3⋯N2^ii^	0.93	2.57	3.439 (3)	155

Symmetry codes: (i) 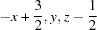; (ii) 
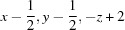
; (iii) 
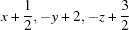
.

**Fig. 1.**
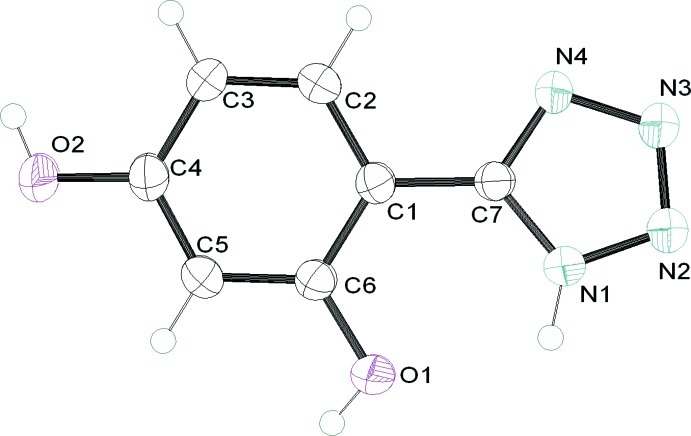
Molecular structure of the title compound, showing the atom-labeling scheme. Displacement ellipsoids are drawn at the 50% probability level.

**Fig. 2.**
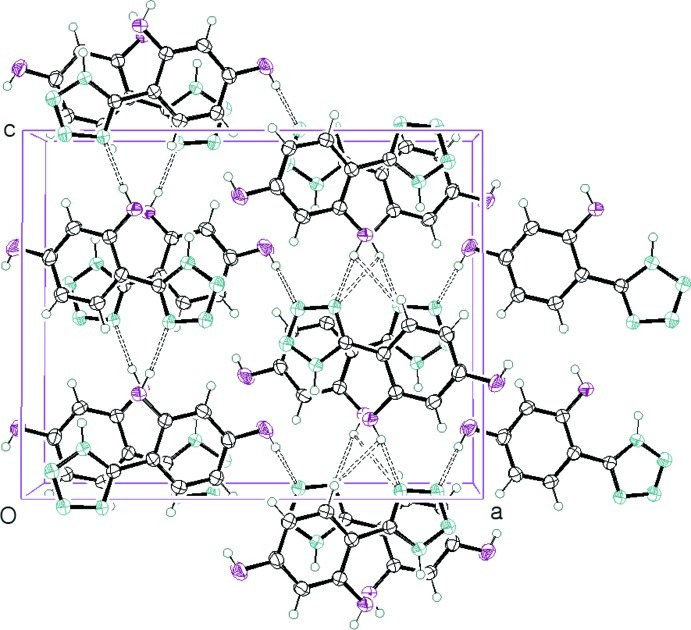
Hydrogen bonding network viewed along the b-axis.

## supplementary crystallographic information

### Comment

The similar structure of the title compound with two tetrazolylbenzenediol molecules per asymmetric unit together with three water molecules was reported in the literature (Gallardo *et al.*, 1995). In this compound, the two tetrazolylbenzendiol are linked through a hydrogen-bonded network to water molecules, forming layers extending along the face of the unit cell (Gallardo *et al.*, 1995). Herein, we report the related X-ray structure of the title compound (I) with eight tetrazolylbenzenediol molecules in unit cell and only one molecule in the asymmetric unit without any solvents. The title compound (I) could be isolated in more than 80% yield *via* the previously reported method (Meyer *et al.*, 1998). Like the structure of related compound (Gallardo *et al.*, 1995), the phenyl ring and the tetrazole ring of each molecule in the asymmetric unit are coplanar (Fig. 1), with the dihedral angle between the benzene and tetrazole rings of 8.45 (13)°. They are connected by a network of intermolecular hydrogen bonds (Fig. 2).

### Experimental

The title compound could be synthesized by the previously reported method (Meyer *et al.*, 1998). Crystal of the title compound suitable for X-ray analysis were grown in ethanol by slow evaporation.

### Refinement

All H atoms were placed in geometrically calculated positions and refined using a riding model with C—H = 0.93, O—H = 0.82 and N—H = 0.89 with *U*_iso_(H) = 1.2Ueq(C), *U*_iso_(H) = 1.5Ueq(O) and *U*_iso_(H) = 1.4Ueq(N).

### Crystal data

**Table:**

C_7_H_6_N_4_O_2_	*D*_x_ = 1.565 Mg m^−^^3^
*M**_r_* = 178.16	Mo *K*α radiation, λ = 0.71073 Å
Orthorhombic, *P**c**c**n*	Cell parameters from 3776 reflections
*a* = 16.109 (2) Å	θ = 3.1–27.9°
*b* = 7.2931 (11) Å	µ = 0.12 mm^−^^1^
*c* = 12.8708 (17) Å	*T* = 296 K
*V* = 1512.2 (4) Å^3^	Block, white
*Z* = 8	0.10 × 0.10 × 0.08 mm
*F*(000) = 736	

### Data collection

**Table:**

Bruker APEXII CCD diffractometer	2452 independent reflections
Radiation source: fine-focus sealed tube	1273 reflections with *I* > 2σ(*I*)
Graphite monochromator	*R*_int_ = 0.110
phi and ω scans	θ_max_ = 31.3°, θ_min_ = 2.5°
Absorption correction: multi-scan (*SADABS*; Bruker, 2006)	*h* = −23→23
*T*_min_ = 0.96, *T*_max_ = 0.98	*k* = −10→9
20096 measured reflections	*l* = −18→18

### Refinement

**Table:**

Refinement on *F*^2^	Primary atom site location: structure-invariant direct methods
Least-squares matrix: full	Secondary atom site location: difference Fourier map
*R*[*F*^2^ > 2σ(*F*^2^)] = 0.057	Hydrogen site location: inferred from neighbouring sites
*wR*(*F*^2^) = 0.168	H atoms treated by a mixture of independent and constrained refinement
*S* = 1.05	*w* = 1/[σ^2^(*F*_o_^2^) + (0.0419*P*)^2^ + 1.9684*P*] where *P* = (*F*_o_^2^ + 2*F*_c_^2^)/3
2452 reflections	(Δ/σ)_max_ < 0.001
124 parameters	Δρ_max_ = 0.35 e Å^−^^3^
0 restraints	Δρ_min_ = −0.26 e Å^−^^3^

### Special details

**Table:**

Geometry. All e.s.d.'s (except the e.s.d. in the dihedral angle between two l.s. planes) are estimated using the full covariance matrix. The cell e.s.d.'s are taken into account individually in the estimation of e.s.d.'s in distances, angles and torsion angles; correlations between e.s.d.'s in cell parameters are only used when they are defined by crystal symmetry. An approximate (isotropic) treatment of cell e.s.d.'s is used for estimating e.s.d.'s involving l.s. planes.
Refinement. Refinement of *F*^2^ against ALL reflections. The weighted *R*-factor *wR* and goodness of fit *S* are based on *F*^2^, conventional *R*-factors *R* are based on *F*, with *F* set to zero for negative *F*^2^. The threshold expression of *F*^2^ > σ(*F*^2^) is used only for calculating *R*-factors(gt) *etc*. and is not relevant to the choice of reflections for refinement. *R*-factors based on *F*^2^ are statistically about twice as large as those based on *F*, and *R*- factors based on ALL data will be even larger.

### Fractional atomic coordinates and isotropic or equivalent isotropic displacement parameters (Å^2^)

**Table:**

	*x*	*y*	*z*	*U*_iso_*/*U*_eq_	
N1	0.86210 (12)	1.0811 (3)	0.86101 (16)	0.0332 (5)	
H101	0.8653 (17)	1.079 (4)	0.792 (2)	0.047 (9)*	
N2	0.92672 (12)	1.1375 (3)	0.91859 (16)	0.0387 (5)	
N3	0.90167 (12)	1.1289 (3)	1.01390 (16)	0.0384 (5)	
N4	0.82224 (11)	1.0674 (3)	1.01992 (15)	0.0322 (5)	
O1	0.74465 (11)	1.0193 (3)	0.71535 (12)	0.0424 (5)	
H1	0.7208	1.0311	0.6595	0.064*	
O2	0.47535 (11)	0.8148 (3)	0.81787 (15)	0.0546 (6)	
H2	0.4521	0.7686	0.8681	0.082*	
C1	0.71543 (13)	0.9735 (3)	0.89292 (17)	0.0275 (5)	
C2	0.66041 (14)	0.9179 (4)	0.97055 (18)	0.0329 (6)	
H2A	0.6780	0.9178	1.0394	0.040*	
C3	0.58072 (14)	0.8633 (4)	0.94770 (18)	0.0350 (6)	
H3	0.5449	0.8270	1.0005	0.042*	
C4	0.55424 (14)	0.8628 (4)	0.84451 (19)	0.0349 (6)	
C5	0.60837 (15)	0.9128 (4)	0.76517 (19)	0.0355 (6)	
H5	0.5909	0.9091	0.6963	0.043*	
C6	0.68889 (14)	0.9686 (3)	0.78936 (17)	0.0292 (5)	
C7	0.79789 (13)	1.0372 (3)	0.92276 (17)	0.0266 (5)	

### Atomic displacement parameters (Å^2^)

**Table:**

	*U*^11^	*U*^22^	*U*^33^	*U*^12^	*U*^13^	*U*^23^
N1	0.0273 (9)	0.0474 (14)	0.0249 (10)	−0.0028 (9)	0.0009 (8)	0.0013 (9)
N2	0.0284 (10)	0.0561 (15)	0.0318 (11)	−0.0080 (10)	−0.0014 (8)	−0.0014 (10)
N3	0.0274 (10)	0.0547 (15)	0.0330 (11)	−0.0071 (10)	−0.0011 (8)	−0.0014 (10)
N4	0.0229 (9)	0.0453 (13)	0.0284 (10)	−0.0029 (9)	−0.0009 (7)	−0.0006 (9)
O1	0.0314 (8)	0.0715 (14)	0.0243 (8)	−0.0072 (9)	−0.0010 (7)	0.0069 (9)
O2	0.0359 (10)	0.0896 (18)	0.0382 (11)	−0.0275 (11)	−0.0102 (8)	0.0150 (11)
C1	0.0251 (10)	0.0299 (13)	0.0276 (11)	0.0006 (9)	−0.0018 (8)	0.0014 (10)
C2	0.0319 (11)	0.0427 (15)	0.0242 (11)	−0.0024 (11)	−0.0030 (9)	0.0018 (10)
C3	0.0280 (11)	0.0471 (16)	0.0300 (12)	−0.0090 (11)	0.0019 (9)	0.0032 (11)
C4	0.0279 (11)	0.0407 (16)	0.0359 (13)	−0.0057 (11)	−0.0046 (9)	0.0027 (11)
C5	0.0332 (11)	0.0466 (16)	0.0266 (12)	−0.0067 (11)	−0.0057 (9)	0.0038 (11)
C6	0.0270 (10)	0.0339 (13)	0.0268 (11)	0.0003 (10)	−0.0004 (9)	0.0031 (10)
C7	0.0254 (10)	0.0299 (13)	0.0245 (11)	0.0017 (9)	0.0006 (8)	0.0013 (9)

### Geometric parameters (Å, º)

**Table:**

N1—N2	1.342 (3)	C1—C2	1.396 (3)
N1—C7	1.343 (3)	C1—C6	1.400 (3)
N1—H101	0.89 (3)	C1—C7	1.459 (3)
N2—N3	1.293 (3)	C2—C3	1.376 (3)
N3—N4	1.358 (3)	C2—H2A	0.9300
N4—C7	1.329 (3)	C3—C4	1.395 (3)
O1—C6	1.360 (3)	C3—H3	0.9300
O1—H1	0.8200	C4—C5	1.391 (3)
O2—C4	1.362 (3)	C5—C6	1.395 (3)
O2—H2	0.8200	C5—H5	0.9300
			
N2—N1—C7	110.1 (2)	C2—C3—H3	120.4
N2—N1—H101	120.8 (18)	C4—C3—H3	120.4
C7—N1—H101	129.1 (18)	O2—C4—C5	117.9 (2)
N3—N2—N1	105.49 (19)	O2—C4—C3	121.7 (2)
N2—N3—N4	111.35 (19)	C5—C4—C3	120.4 (2)
C7—N4—N3	106.20 (18)	C4—C5—C6	119.7 (2)
C6—O1—H1	109.5	C4—C5—H5	120.2
C4—O2—H2	109.5	C6—C5—H5	120.2
C2—C1—C6	118.7 (2)	O1—C6—C5	122.5 (2)
C2—C1—C7	118.8 (2)	O1—C6—C1	117.3 (2)
C6—C1—C7	122.5 (2)	C5—C6—C1	120.3 (2)
C3—C2—C1	121.6 (2)	N4—C7—N1	106.87 (19)
C3—C2—H2A	119.2	N4—C7—C1	124.7 (2)
C1—C2—H2A	119.2	N1—C7—C1	128.4 (2)
C2—C3—C4	119.3 (2)		

### Hydrogen-bond geometry (Å, º)

**Table:**

*D*—H···*A*	*D*—H	H···*A*	*D*···*A*	*D*—H···*A*
O1—H1···N4^i^	0.82	1.94	2.759 (3)	173
O2—H2···N3^ii^	0.82	2.00	2.817 (3)	173
N1—H101···O1	0.89 (3)	2.22 (3)	2.701 (3)	113 (2)
N1—H101···O2^iii^	0.89 (3)	2.40 (3)	3.034 (3)	129 (2)
C3—H3···N2^ii^	0.93	2.57	3.439 (3)	155

Symmetry codes: (i) −*x*+3/2, *y*, *z*−1/2; (ii) *x*−1/2, *y*−1/2, −*z*+2; (iii) *x*+1/2, −*y*+2, −*z*+3/2.

## References

[bb1] Bruker (2006). *APEX2*, *SAINT* and *SADABS* Bruker AXS Inc., Madison, Wisconsin, USA.

[bb2] Farrugia, L. J. (2012). *J. Appl. Cryst.* **45**, 849–854.

[bb3] Gallardo, H., Meyer, E. & Vencato, I. (1995). *Acta Cryst.* C**51**, 2430–2432.

[bb4] Meyer, E., Zucco, C. & Gallardo, H. (1998). *J. Mater. Chem.*, **8**, 1351–1354.

[bb5] Sheldrick, G. M. (2008). *Acta Cryst.* A**64**, 112–122. 10.1107/S0108767307043930 18156677

